# Multiple P2Y receptors couple to calcium-dependent, chloride channels in smooth muscle cells of the rat pulmonary artery

**DOI:** 10.1186/1465-9921-6-124

**Published:** 2005-10-26

**Authors:** Krongkarn Chootip, Alison M Gurney, Charles Kennedy

**Affiliations:** 1 Department of Physiology and Pharmacology, University of Strathclyde, Strathclyde Institute for Biomedical Sciences, John Arbuthnott Building, 27 Taylor Street, Glasgow G4 ONR, UK; 2 Department of Physiology, Faculty of Medical Science, Naresuan University, Phitsanulok 65000, Thailand

## Abstract

**Background:**

Uridine 5'-triphosphate (UTP) and uridine 5'-diphosphate (UDP) act via P2Y receptors to evoke contraction of rat pulmonary arteries, whilst adenosine 5'-triphosphate (ATP) acts via P2X and P2Y receptors. Pharmacological characterisation of these receptors in intact arteries is complicated by release and extracellular metabolism of nucleotides, so the aim of this study was to characterise the P2Y receptors under conditions that minimise these problems.

**Methods:**

The perforated-patch clamp technique was used to record the Ca^2+^-dependent, Cl^- ^current (I_Cl,Ca_) activated by P2Y receptor agonists in acutely dissociated smooth muscle cells of rat small (SPA) and large (LPA) intrapulmonary arteries, held at -50 mV. Contractions to ATP were measured in isolated muscle rings. Data were compared by Student's t test or one way ANOVA.

**Results:**

ATP, UTP and UDP (10^-4^M) evoked oscillating, inward currents (peak = 13–727 pA) in 71–93% of cells. The first current was usually the largest and in the SPA the response to ATP was significantly greater than those to UTP or UDP (P < 0.05). Subsequent currents tended to decrease in amplitude, with a variable time-course, to a level that was significantly smaller for ATP (P < 0.05), UTP (P < 0.001) and UDP (P < 0.05) in the SPA. The frequency of oscillations was similar for each agonist (mean≈6–11.min^-1^) and changed little during agonist application. The non-selective P2 receptor antagonist suramin (10^-4^M) abolished currents evoked by ATP in SPA (n = 4) and LPA (n = 4), but pyridoxalphosphate-6-azophenyl-2',4'-disulphonic acid (PPADS) (10^-4^M), also a non-selective P2 antagonist, had no effect (n = 4, 5 respectively). Currents elicited by UTP (n = 37) or UDP (n = 14) were unaffected by either antagonist. Contractions of SPA evoked by ATP were partially inhibited by PPADS (n = 4) and abolished by suramin (n = 5). Both antagonists abolished the contractions in LPA.

**Conclusion:**

At least two P2Y subtypes couple to I_Cl,Ca _in smooth muscle cells of rat SPA and LPA, with no apparent regional variation in their distribution. The suramin-sensitive, PPADS-resistant site activated by ATP most resembles the P2Y_11 _receptor. However, the suramin- and PPADS-insensitive receptor activated by UTP and UDP does not correspond to any of the known P2Y subtypes. These receptors likely play a significant role in nucleotide-induced vasoconstriction.

## Background

Uridine 5'-triphosphate (UTP) and uridine 5'-diphosphate (UDP) act via P2Y receptors, whilst adenosine 5'-triphosphate (ATP) acts via P2X as well as P2Y receptors, to modulate vascular tone [1-3]. P2X receptors are ligand-gated cation channels and the ability of the P2X_1 _subtype to mediate rapid, transient inward currents in pulmonary artery smooth muscle cells [4,5] and induce constriction of the pulmonary vasculature (see [6] and references therein) has been characterised in some depth. P2Y receptors are G protein-coupled receptors and P2Y agonists act at smooth muscle receptors to evoke vasoconstriction in the rat perfused lung at resting tone, but induce vasodilation via endothelial receptors if muscle tone is first raised [7-10]. Similarly, P2Y agonists are contractile at resting tone and relaxant at raised tone in isolated branches of rat intrapulmonary arteries [11-13]. Compared with P2X receptors much less is known about which of the eight mammalian P2Y subtypes (P2Y_1,2,4,6,11,12,13,14_) [14,15] are expressed in pulmonary vascular smooth muscle or about the signalling pathways through which they act.

In a previous study [6] we showed that UTP and UDP both act via two P2Y receptors to evoke contraction of rat isolated pulmonary arteries. For each agonist one site was insensitive to the antagonists suramin and pyridoxalphosphate-6-azophenyl-2',4'-disulphonic acid (PPADS), whilst the other was inhibited by suramin, but not PPADS. UTP is a potent agonist at the P2Y_2 _and P2Y_4 _receptors and a weaker agonist at the P2Y_6 _subtype [16,17]. Of these three receptors, only the P2Y_2 _is suramin-sensitive and PPADS-insensitive [18], so this is likely to be one of the sites of action of UTP. The molecular identity of the suramin-and PPADS-insensitive site of action of UTP is unclear as the P2Y_4 _and P2Y_6 _subtypes are both reported to be antagonised by PPADS, but not suramin [18-20]. UDP is a potent agonist at the P2Y_6 _receptor only [16,17]. mRNA for this subtype and suramin-insensitive contractions to UDP in pulmonary arteries have been demonstrated [12], but the lack of effect of PPADS against the contractions evoked by UDP in our previous study are inconsistent with the P2Y_6 _receptor.

A number of factors that can complicate the characterisation of P2Y receptors may have prevented the clear identification of the P2Y receptors mediating the contractions seen in previous studies. These include the release of nucleotides from cells, their breakdown by ecto-nucleotidases and their bioconversion by ecto-nucleoside diphosphokinase (eNDPK) [16,21-24]. Thus, as well as a direct action at the P2Y_6 _receptor, UTP can also act indirectly, after dephosphorylation to UDP. Likewise, UDP can be converted to UTP by eNDPK and so act indirectly at P2Y_2 _and P2Y_4 _receptors. At present, potent and selective inhibitors of the ecto-enzymes are not available, but one way to minimise these metabolic problems is to apply the agonists to rapidly perfused, dissociated cells.

The aim of the present study was to extend the pharamacological characterisation of the P2Y receptors mediating pulmonary vasoconstriction, in conditions that minimise the influence of the release and extracellular metabolism of nucleotides. We used the perforated-patch clamp technique to record the Ca^2+^-dependent, Cl^- ^current (I_Cl,Ca_) induced by nucleotides in single, acutely dissociated pulmonary artery smooth muscle cells [4,5]. The pulmonary vascular bed has well characterised regional differences in receptor and ion channel distribution, including I_Cl,Ca _[8,25], so the responses of cells isolated from large and small pulmonary arteries were compared. In addition to UTP and UDP, we also applied ATP, an agonist at the P2Y_1,2,4,11 & 12 _subtypes [14] and determined the ability of the P2 antagonists suramin and PPADS to inhibit the responses evoked by each of the agonists. We found that at least two P2Y subtypes couple to I_Cl,Ca_, with no apparent regional variation in their distribution. The suramin-sensitive, PPADS-resistant site activated by ATP most resembles the P2Y_11 _receptor. However, the suramin- and PPADS-insensitive receptor activated by UTP and UDP does not correspond to any of the known P2Y subtypes.

## Methods

### Isolated cell preparation

Male Sprague-Dawley rats (150 – 250 g) were killed by the approved Schedule 1 method of cervical dislocation and exsanguination. After thoracotomy, the heart and lungs were removed *en bloc*, the lungs separated and small (SPA, 200–500 μm id) and large (LPA, 1.0–1.5 mm id) intrapulmonary arteries dissected out. The arteries were cut open longitudinally and strips of smooth muscle bathed in a dissociation medium (DM) composed of (mM); NaCl 110; KCl 5; KH_2_PO_4 _0.5; NaH_2_PO_4 _0.5; NaHCO_3 _10; N-[2-hydroxyethyl]piperazine-N'-[2-ethane-sulfonic acid] (HEPES)10; phenol red 0.03; taurine 10; ethylenediaminetetraacetic acid (EDTA) 0.5; MgCl_2 _2; glucose 10 and CaCl_2 _0.16, titrated to pH 7.0 with KOH. After incubation in DM containing 0.6 – 0.8 mg.ml^-1 ^papain, 0.04% BSA and 0.4 mM dithiothreitol at 37°C (15 min for LPA, 10 min for SPA), collagenase (0.6 – 0.8 mg.ml^-1^; type IA) was added and the tissues incubated for a further 10 (LPA) or 5 (SPA) min. Cells were then dispersed by mild trituration in enzyme-free solution and used within 7 hours.

### Electrophysiological recording

Cells were placed in a 50 μl chamber and superfused at room temperature with physiological salt solution (PSS) composed of (mM): NaCl 122; KCl 5; HEPES 10; KH_2_PO_4 _0.5; NaH_2_PO_4 _0.5; MgCl_2 _1; glucose 11; CaCl_2 _1.8; titrated to pH 7.3 with NaOH. Electrophysiological responses of isolated smooth muscle cells were studied in the whole-cell, perforated-patch mode with amphotericin B (150 μg.ml^-1^) added to a pipette solution of the following composition (mM): KCl 125; MgCl_2 _4; HEPES 10; ethylene glycol-bis(2-aminoethylether)-N,N,N',N'-tetraacetic acid (EGTA) 0.02, titrated to pH 7.3 with KOH. Pipette resistance was 4–8 MΩ. The cells were voltage-clamped at -50 mV using an Axopatch 200A amplifier (Axon Instruments). Data were recorded and analysed with a personal computer interfaced with a Digidata 1200 A/D converter (Axon Instruments) using Axotape and pClamp (V5) software (Axon Instruments). Current responses to -10 mV hyperpolarizing steps were used to measure cell capacitance.

We have reported previously that 10^-4 ^M ATP, UTP and UDP each evoked pronounced contractions of rat isolated SPA and LPA and that 10^-4 ^M suramin and PPADS produced maximum inhibition of these responses [6]. Therefore, this concentration of these drugs was used here. All were applied to the cells using a gravity-feed perfusion system, for which the time for complete solution exchange was less than 2 s. Only one agonist was applied to each cell. P2Y receptor-mediated contractions develop slowly and take 5–10 min to reach a steady-state plateau, therefore, in most cases the agonists were applied to the cells for 5 min or more.

### Electrophysiological analysis

The rat pulmonary artery is a relatively short vessel, with only a thin layer of smooth muscle cells. Enzymatic dissociation produces a lower yield of cells that are smaller and often less robust than those from systemic blood vessels. Consequently, although oscillating inward currents were observed in response to P2Y receptor agonists in the majority of cells studied, quantitative analysis was hampered by the short period of time that many cells could be maintained in the perforated-patch configuration or by the disappearance of the response during the recording. Quantitative analysis was applied only to cells that could be held for 5 min or more and in which the oscillating currents lasted more than 4 min. In these cells the following parameters were measured: a) the peak amplitude of each current (pA), which was normalised against the cell capacitance (pF) to control for variations in cell size; b) the rise time (ms) of the current at each oscillation from baseline holding current to peak; c) the width of each oscillation (ms) at the point where it reached 50% of its peak amplitude. For each, the average value during successive 30 s intervals over a 4 min period was calculated and compared. Finally, the frequency of oscillations (peak.min^-1^) was measured as the number of transient currents occurring during successive 1 min intervals over a 4 min period.

To investigate the effects of P2 receptor antagonists on the currents, some cells were preincubated with antagonist for 5 min before adding an agonist, but in most cases an agonist was applied for 2 min and then suramin or PPADS were co-applied for a further 2–3 min. The current amplitude and frequency were measured and average values compared for the 1 min periods immediately before and after antagonist addition. The data were compared with control cells where agonist alone was added and the currents measured over the same time course.

### Tension recording

Rat SPA and LPA were dissected out as described above, cut into rings 5 mm long and mounted horizontally in 1 ml baths on a pair of intraluminal wires [6]. Tissues were allowed to equilibrate under a resting tension of 0.5 g (SPA) and 1.0 g (LPA) for 60 min at 37°C in PSS. Tension was recorded with Grass FT03 isometric force transducers connected to a MacLab/4e system, using Chart 3.3 software (AD Instruments). Cumulative concentration-response curves to ATP were obtained in rings in the absence of antagonist (control) or in the presence of a single concentration (3 × 10^-5^, 10^-4 ^or 3 × 10^-4 ^M) of suramin or PPADS. Contractions generally took 1–4 min to reach a plateau and are expressed as a percentage of the contraction induced in the same preparation by 4 × 10^-2 ^M KCl, which was applied by replacement of the PSS solution with PSS in which the KCl concentration was raised by equimolar substitution for NaCl.

### Data analysis

Values in the text and figures refer to mean ± S.E.M.. Data were compared by paired and unpaired t-tests, or one-way analysis of variance and Tukey's comparison as appropriate. Differences were considered significant when P < 0.05.

### Drugs and solutions

ATP (magnesium salt), UDP (sodium salt), UTP (sodium salt), suramin hexasodium and PPADS tetrasodium (Sigma/RBI, UK) were dissolved in deionised water as 100 mM stock solutions and diluted in PSS before application to the cells.

## Results

### P2Y receptor agonists induce oscillating inward currents

**Figure 1:**
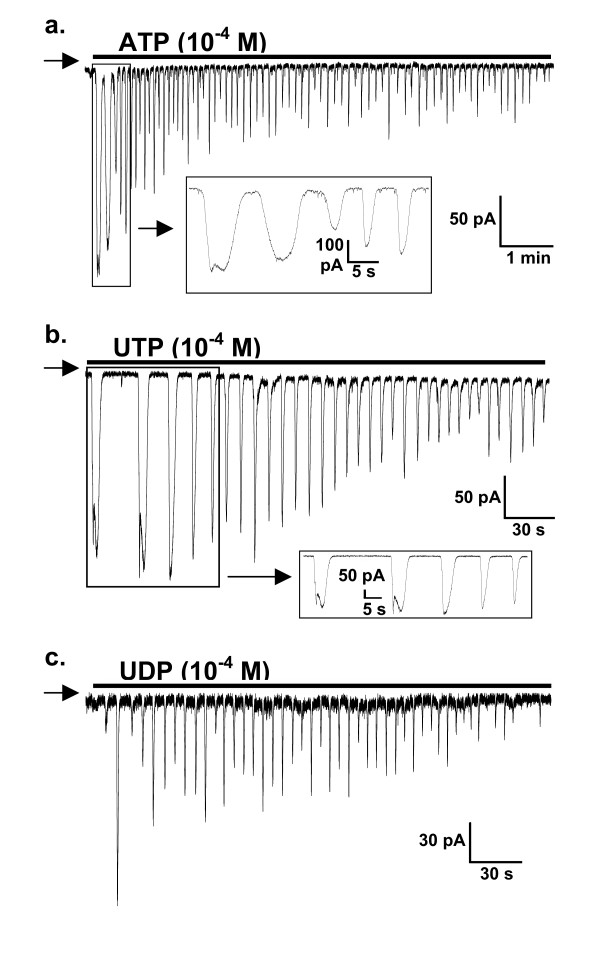
**Oscillating currents induced by P2Y receptor agonists**. (a) ATP, (b) UTP and (c) UDP (all 10^-4 ^M), added as indicated by the horizontal bars, evoked oscillating inward currents in smooth muscle cells isolated from SPA (a & c) and LPA (b) and voltage-clamped at -50 mV. The insets show W-shape currents induced by (a) ATP and (b) UTP. The arrow on the left-hand side of each trace indicates zero holding current.

**Figure 2:**
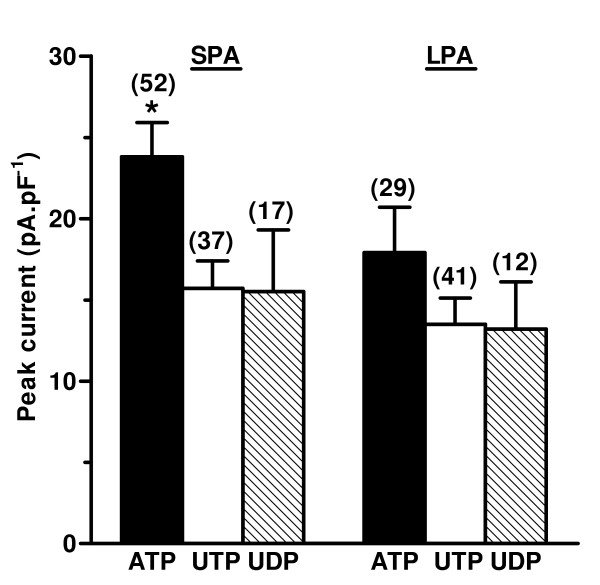
**Amplitude of the first inward current induced by P2Y receptor agonists**. The mean ± s.e.mean of the peak amplitude of the initial inward currents evoked by 10^-4 ^M ATP, UTP and UDP in SPA and LPA isolated smooth muscle cells, voltage-clamped at -50 mV, are shown. The number of cells for each is indicated in parentheses. *P < 0.05 for responses to ATP versus UTP and UDP in SPA.

ATP, UTP and UDP (10^-4 ^M) each evoked inward currents (peak amplitude = 13 – 727 pA) in most SPA (n = 118) and LPA (n = 117) smooth muscle cells held at -50 mV (ATP-91%/88%, UTP-91%/93%, UDP-71%/81%, SPA/LPA respectively). Outward currents or no response were evoked in the remaining cells, which were not studied further. In most cells the inward currents activated in an oscillating manner (Figure 1). The first current was usually the largest and subsequent currents decreased in amplitude, with a variable time-course. The peak of the first current appeared to be larger for ATP than UTP or UDP (Figure 2), but this was significant only in the SPA (P < 0.05). For each agonist there was no significant difference in the amplitude of the first response between the small and large vessels. The initial current often had a "W-shaped" profile (Figure 1), but in most cells (85%) the biphasic profile disappeared by the second, third or fourth oscillation, such that subsequent currents were monophasic. ATP, UTP and UDP each evoked this profile of responses in a similar proportion of cells.

### Quantitative analysis of oscillating currents

The decrease in the amplitude of the oscillating currents during agonist application complicated the quantification of the effects of P2Y antagonists, so it was necessary to first quantify the time-course of the currents. In order to be able to study the effects of both an agonist and antagonist on the same cell, the analysis was limited to a subpopulation of cells that were maintained under voltage-clamp for more than 5 min and in which the oscillating currents lasted for 4 min or more.

**Figure 3:**
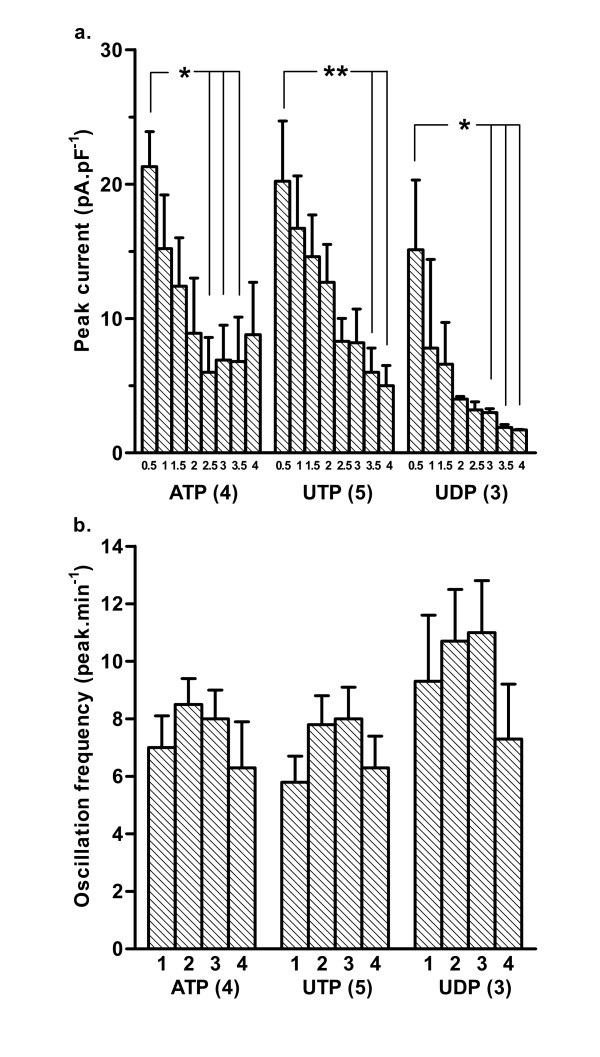
**The amplitude and frequency of oscillating currents induced by P2Y receptor agonists**. (a) The mean amplitude of oscillating currents induced by ATP, UTP and UDP (10^-4 ^M) was measured over successive 30 s intervals for 4 min in smooth muscle cells isolated from SPA and voltage-clamped at -50 mV. 0.5 = the first 30 s of agonist application, 1 = 30 – 60 s, and so on. (b) The frequency of the oscillations in the same cells was measured over successive 1 min intervals. 1 = the first min of agonist application, 2 = the second min, and so on. Vertical bars represent s.e.mean. The number of cells for each agonist is shown in parentheses. * P < 0.05 for amplitude of peak current at 30 s versus that at 2:30, 3:00, 3:30 and 4:00 min; ** P < 0.01 for 30 s versus 3:30 and 4:00 min.

In both SPA (Figure 3a) and LPA (not shown), the amplitude of the oscillating currents tended to decrease over successive 30 s intervals, particularly within the first 2 min, and this was significant for ATP (P < 0.05), UTP (P < 0.01) and UDP (P < 0.05) in the SPA. The oscillations induced by ATP, UTP and UDP had similar frequencies (mean≈6–11.min^-1^), both in SPA (Figure 3b) and LPA (not shown) and showed no significant change over 4 min, apart from a small increase in the LPA between the first and second min after UTP application (P < 0.05). The currents evoked by ATP in the first 30 s had a rise time of 1.7 ± 0.3 s and width at 50% peak of 2.9 ± 0.4 s (n = 4) in SPA and 1.5 ± 0.6 s and 2.2 ± 0.3 s (n = 5) respectively in LPA. The rise time and width at 50% peak then decreased significantly (P < 0.01) over the next 60–90 s to a steady state of around 0.8 s in both SPA and LPA. The width at 50% peak of currents evoked by UTP in the first 30 s was 2.1 ± 0.5 s (n = 5) in SPA and 2.1 ± 0.5 s (n = 5) in LPA and both decreased significantly (P < 0.01) over the next 60–90 s to a steady state of also about 0.8 s. In contrast, currents evoked by UTP and UDP in SPA and LPA showed no significant change in rise time and those to UDP in SPA and LPA showed no significant change in width at 50% peak, all having a steady-state value of about 0.8 s.

### Effects of P2 receptor antagonists

Having quantified the time-course of the agonist-induced oscillating currents, we then determined the effects of the P2Y antagonists suramin and PPADS (10^-4 ^M). In most cells currents were initiated by an agonist, the antagonist was then coapplied and the currents compared for 1 min before and after antagonist addition. To take into account the decline in current amplitude normally seen over this time-course (~20–40%), the % decrease in amplitude over the 2 analysis periods was calculated and compared with that in control cells where agonist alone was added.

**Figure 4:**
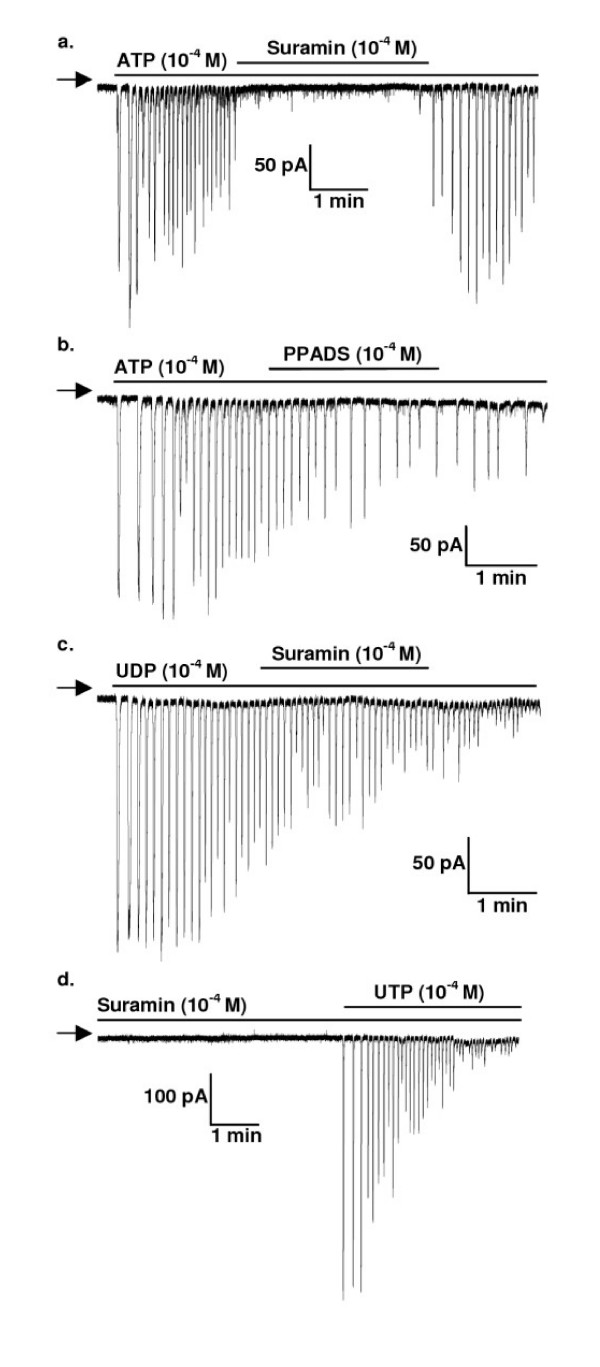
**The effects of P2 receptor antagonists on oscillating currents**. The P2 receptor antagonists suramin and PPADS (10^-4 ^M) were applied either 2 min after oscillations were induced by continuous application of 10^-4 ^M (a, b) ATP or (c) UDP or (d) 5 min before application of 10^-4 ^M UTP to SPA (a, b) or LPA (c, d) dissociated smooth muscle cells voltage-clamped at -50 mV. The horizontal bars indicate agonist and antagonist applications. The arrow on the left-hand side of each trace indicates zero holding current.

**Figure 5:**
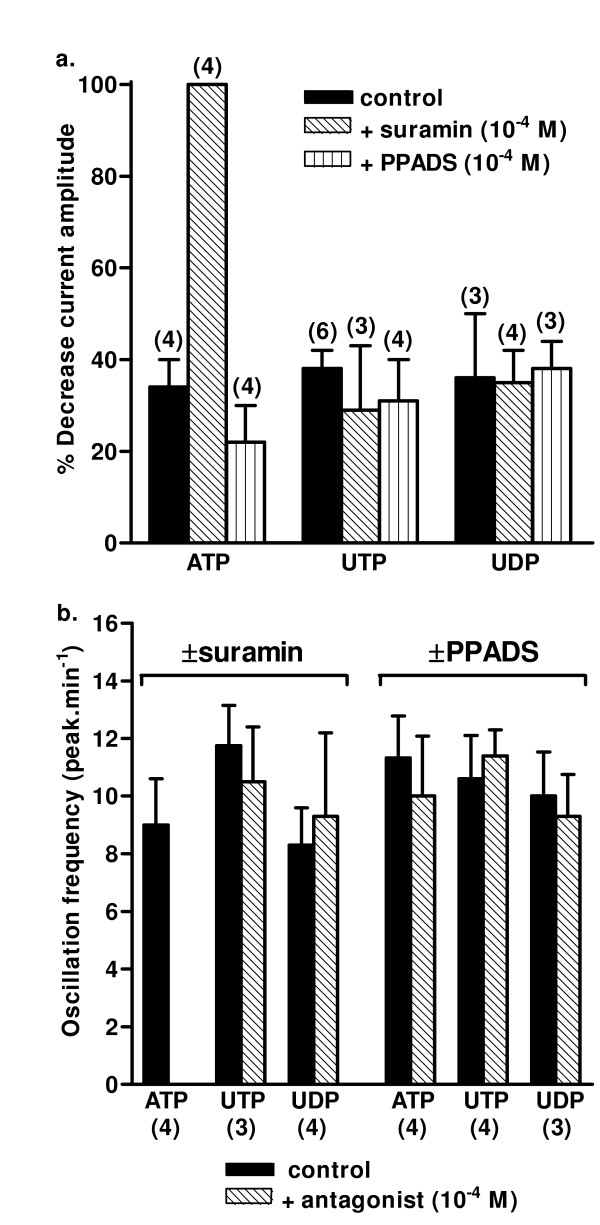
**The effects of P2 receptor antagonists on oscillating current amplitude and frequency**. The effects of suramin and PPADS (10^-4 ^M) on (a) the amplitude and (b) the frequency of oscillating inward currents induced by ATP, UTP and UDP (10^-4 ^M) in SPA dissociated smooth muscle cells, voltage-clamped at -50 mV, are shown. The agonist was applied for 2 min and then suramin or PPADS was coapplied for a further 2–3 min. The current amplitude was measured for 1 min immediately before and after antagonist addition and average values calculated. The % decrease in amplitude was then calculated as the difference in the 2 average values. The control data were obtained over the same time-course in cells where agonist alone was added. The average frequency of oscillations was also measured for 1 min immediately before and after antagonist addition and compared directly. Vertical lines show s.e.mean. The number of cells is shown in parentheses.

Suramin rapidly and reversibly abolished the currents evoked by ATP in SPA (Figure 4a, 5) and LPA (not shown). Additionally, ATP did not elicit currents if cells were preincubated with suramin for 5 min (n = 2, not shown). In contrast, PPADS (10^-4 ^M) had no effect on the amplitude or frequency of the ATP-induced currents in SPA (Figure 4b, 5) or LPA (not shown). The rise-time and width at 50% peak were also unaffected (not shown). PPADS was also ineffective if applied for 5 min prior to ATP (n = 2). Neither suramin nor PPADS had any effect on the amplitude or frequency of the oscillating currents elicited by UTP or UDP in either SPA or LPA (Figure 4c, 5). The rise-time and width at 50% peak were also unaffected (not shown). PPADS (n = 12) and suramin (n = 7) (Figure 4d) were also ineffective if applied for 5 min before UTP.

### Effects of suramin and PPADS on contractions evoked by ATP

**Figure 6:**
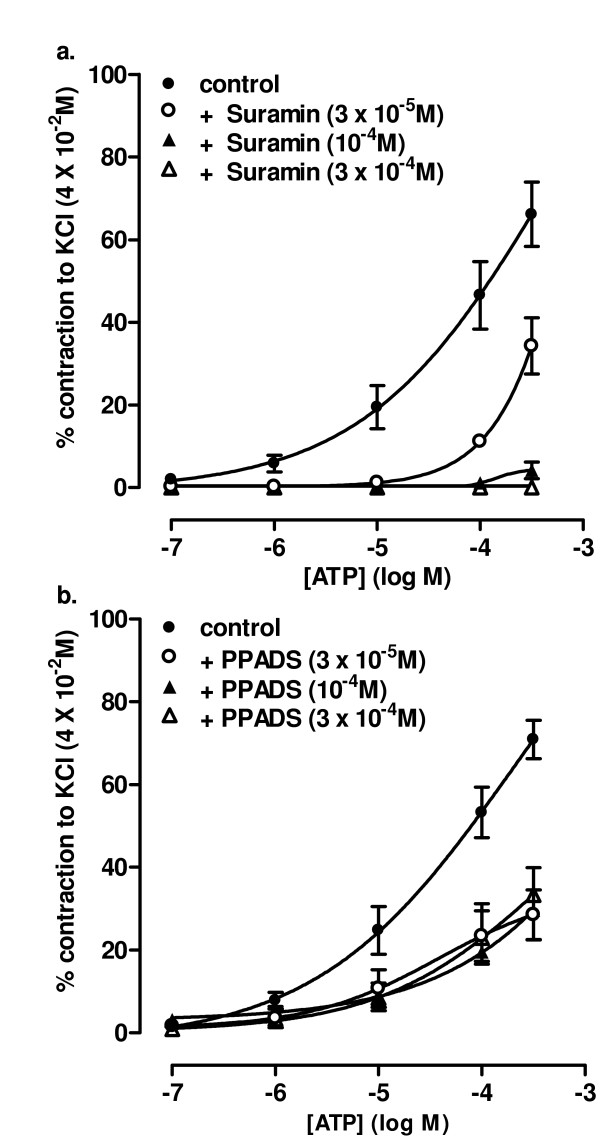
**Effects of suramin and PPADS on contractions evoked by ATP**. The effects of (a) suramin and (b) PPADS on contractions of rat isolated SPA induced by ATP are shown. Cumulative concentration-response curves to ATP (10^-7 ^- 3 × 10^-4 ^M) were obtained in rings in the absence of antagonist (control) or in the presence of 3 × 10^-5^, 10^-4 ^or 3 × 10^-4 ^M of antagonist. Contractions are expressed as a percentage of the contraction induced by 4 × 10^-2 ^M KCl. Vertical lines show s.e.mean. n = 5 for suramin and 4 for PPADS.

We have reported previously the effects of suramin and PPADS on contractions of rat pulmonary arteries induced by UTP and UDP [6]. Since suramin abolished current oscillations induced by ATP, but not UTP or UDP, we investigated if nucleotide-induced contractions showed the same differential sensitivity. We report that ATP (10^-7 ^- 3 × 10^-4 ^M) evoked concentration-dependent contractions of the rat SPA (Figure 6). Suramin (3 × 10^-5 ^- 10^-4 ^M) caused a progressive rightward shift of the ATP concentration-response curve and the responses were abolished by the highest concentration of the antagonist (Figure 6a). PPADS (3 × 10^-5 ^M) also shifted the ATP concentration-response curve to the right, but increasing its concentration to 10^-4 ^M and 3 × 10^-4 ^M) produced no further inhibition (Figure 6b). In rat LPA ATP induced contractions only at 10^-4 ^M and above [6] and these small contractions were abolished by 3 × 10^-4 ^M suramin or PPADS (not shown).

## Discussion

The present study shows that ATP, UTP and UDP induce oscillating inward currents with similar amplitudes and frequencies in smooth muscle cells of rat pulmonary arteries. Such Cl^- ^currents have been reported previously in these cells and are dependent upon nucleotide-evoked release of Ca^2+ ^from sarcoplasmic reticulum stores [4,5,26]. The P2Y_1_, P2Y_2_, P2Y_4_, P2Y_6 _and P2Y_11 _receptors all couple to the G_q/11 _G proteins, leading to the release of IP_3_-sensitive Ca^2+ ^stores [14] and so could, if present in the tissue, mediate activation of I_Cl,Ca_. UTP and UDP both acted at a site that was insensitive to the antagonists suramin and PPADS, which may be the P2Y_6 _receptor or perhaps a novel receptor. ATP clearly acted via a different subtype, which most resembles the P2Y_11 _receptor. There were few differences apparent between the SPA and LPA, consistent with our previous conclusion from contractile studies that there is no regional variation in the P2Y subtype distribution. Thus, multiple subtypes of P2Y receptor are widely expressed in pulmonary artery smooth muscle and are likely to play a role in nucleotide-induced vasoconstriction.

### P2Y receptors in SPA and LPA

In these experiments, the currents evoked by ATP in cells from the SPA and LPA were abolished by suramin, but unaffected by PPADS. ATP is an agonist at the P2Y_1,2,4 & 11 _receptors [15] and the P2Y_1 _and P2Y_4 _subtypes can be ruled out, because PPADS antagonises both of these [4]. The P2Y_2 _receptor can also be discounted as responses to the P2Y_2 _agonist UTP were not inhibited by suramin. The remaining P2Y_11 _receptor is antagonised by suramin, but not PPADS [27], consistent with a role in mediating the ATP-induced I_Cl,Ca_. This is problematic, however, as the rat P2Y_11 _receptor has yet to be cloned. Indeed, it is not clear that it is present in the rodent genome, although a previous pharmacological study is also consistent with its expression in rat blood vessels [28]. Further studies are required to address this issue. Note that ATP has been reported to be an agonist at the P2Y_12 _receptor [29] and that a contractile P2Y_12 _receptor was recently reported in human blood vessels [30]. However, the agonist action of ATP has been questioned [31] and the P2Y_12 _receptor couples to G_i _and so is unlikely to induce the release of Ca^2+ ^stores needed to activate I_Cl,Ca _in rat pulmonary arteries [5].

UTP and UDP also activated I_Cl,Ca_, but the responses were unaffected by suramin or PPADS. This is consistent with the lack of effect of the antagonists on UTP- and UDP-induced vasoconstriction in the rat perfused pulmonary vascular bed [10] and with the antagonist-insensitive component of UTP and UDP contraction of isolated pulmonary artery [6], but contrasts with the abolition by suramin of UTP-elicited oscillating currents seen previously in single cells [5,12]. The reason for these differences in suramin activity is not clear. Which receptor(s) mediated the actions of UTP and UDP in the present study is also unclear. UTP is an agonist at the P2Y_2_,_4 _&_6 _subtypes, whilst UDP is only active at the P2Y_6 _receptor [2,15]. Detailed studies show clearly that the P2Y_2 _receptor is antagonised by suramin and the P2Y_4 _receptor by PPADS (2,20). So, these subtypes do not mediate the effects of UTP (or UDP) seen here.

If the P2Y_2 _and P2Y_4 _receptors are ruled out, then the P2Y_6 _receptor is the prime candidate for the site of action of UTP and UDP. Indeed, its mRNA is present in rat pulmonary artery smooth muscle and it has been proposed to underlie the UDP-induced I_Cl,Ca _[12]. However, the effects of suramin and PPADS at this site are not well characterised. In the only study on the cloned rat P2Y_6 _receptor, 10^-4 ^M suramin (the same concentration used in the present study) depressed the agonist response by 20% [32]. Similar inhibition (27%) was seen at the cloned human P2Y_6 _receptor [18]. PPADS was not tested at the rat receptor, but at 10^-4 ^M it inhibited the response to UDP at the human subtype by 69%. This pronounced effect of PPADS is inconsistent with the P2Y_6 _receptor being the receptor through which UTP and UDP activated I_Cl,Ca _in the present study. Further characterisation of the effects of suramin and PPADS at the recombinant rat P2Y_6 _receptor is, however, needed to substantiate this conclusion.

If the P2Y_2_, P2Y_4 _and P2Y_6 _receptors are not the site(s) of action of UTP and UDP, then what is? One possibility is that UTP and UDP activated I_Cl,Ca _in rat SPA and LPA smooth muscle via a novel, as yet uncloned P2Y receptor or another, non-P2Y receptor. For example, UDP has been proposed to interact with cysteinyl leukotriene receptors in human mast cells [33,34]. Alternatively, one of the known P2Y receptors may interact with another P2Y subtype, or with a non-P2Y receptor, to form a dimer with novel pharmacological properties. Indeed, the P2Y_1 _and P2Y_2 _receptors both appear to form dimers with the A1 adenosine receptor [35]. Further studies are needed to investigate these possibilities.

### P2X receptors in SPA and LPA

In this study, the currents evoked by ATP, UTP and UDP had similar time-courses, as measured by rise time and width at 50% peak. This may appear surprising as ATP, but not UTP or UDP, is also an agonist at the P2X_1 _receptor and so might be expected to activate an initial, rapid, transient inward current, in addition to the slower, longer lasting, P2Y-mediated oscillations, as has been reported previously in rat pulmonary artery smooth muscle cells [4,5]. The apparent absence of the transient response may be due to the relatively slow speed of application of ATP used here. The P2X_1 _receptor desensitizes rapidly and slow agonist administration elicits much slower and smaller currents in vascular smooth muscle cells [36]. Although this would be disadvantageous if studying P2X receptors, by minimizing the P2X response it is in fact an advantage when P2Y receptors are under study. The initial current evoked by ATP in SPA and LPA may well be a mixture of P2X_1 _and P2Y receptor-induced responses, which would explain the larger amplitude of the initial ATP-induced current, compared with UTP and UDP. Nevertheless, any P2X_1 _component appears to play a relatively minor role and would not contribute to the sustained phase of oscillations.

### Contribution of P2Y subtypes to contractions

Although the receptors that mediate activation of I_Cl,Ca _by nucleotides in the rat pulmonary artery have not been identified unequivocally, we can still consider their role in vasoconstriction of the rat pulmonary vascular bed [10] and isolated arteries [6,12,13]. In this study, contractions of the SPA elicited by ATP were abolished by suramin, but only partially inhibited by PPADS. The PPADS-resistant contractions likely reflect release of Ca^2+ ^stores, causing the I_Cl,Ca _recorded here. They may also involve Ca^2+ ^influx via L-type Ca^2+ ^channels, opened by depolarisation due to I_Cl,Ca_. Further experiments using channel blockers are needed to confirm this. The P2X_1 _receptor in SPA smooth muscle [6] is most likely to underlie the remaining suramin- and PPADS-sensitive component. Interestingly, contractions of the rat LPA were abolished by both suramin and PPADS, suggesting that only one receptor, probably the P2X_1 _receptor, mediates the contractile actions of ATP here. This is consistent with the much lower contractile potency of ATP in LPA [6], but it suggests that the similar suramin- and PPADS-insensitive I_Cl,Ca _observed in response to ATP in LPA and SPA may serve different functions. In our previous study [6] contractions of rat SPA induced by UTP and UDP were not inhibited by PPADS and were only partially suppressed by suramin. These antagonist-resistant contractions again likely reflect release of Ca^2+ ^stores and activation of I_Cl,Ca_. The identity of the suramin-sensitive receptors remains to be determined.

### Advantages of the patch clamp technique

This study shows that recording ion currents in single cells can be useful in characterising the receptors expressed in tissues where multiple subtypes are present. A particular problem with P2Y receptors is ecto-nucleotidases, which are inhibited by PPADS in smooth muscle [37] and other tissues [38,39]. Recording from rapidly perfused, single cells minimises the problems created by extracellular metabolism in whole tissues, which may explain why PPADS potentiated contractions to UTP and UDP in the intact artery [6], but had no effect on activation of I_Cl,Ca _in single cells. Such studies also allow the regional variation in ion channel expression to be studied. Interestingly, we recorded I_Cl,Ca _in a similar proportion of rat SPA and LPA smooth muscle cells, whereas in rabbits it is more predominant in smaller pulmonary arteries [25]. Limitations of the patch clamp technique encountered here were short recording times, wide variation in current amplitude between cells and a decline in the amplitude of I_Cl,Ca _over the recording period, all of which hampered quantitative analysis of antagonist action. It is not clear why rundown occurred, as loss of diffusible cytosolic factors into the recording pipette should have been minimised with the perforated-patch technique. Similar rundown was seen in previous patch clamp studies in these cells [4,5] and with ATP- and UTP-induced oscillations in cytosolic [Ca^2+^] [26]. Thus, the decline in I_Cl,Ca _may in fact reflect a physiological mechanism of signalling whereby the P2Y receptors become desensitised and/or intracellular stores release progressively less Ca^2+ ^during maintained activation of P2Y receptors.

### Conclusion

The results of the present study indicate the presence of at least two different subtypes of P2Y receptors mediating oscillating inward currents in rat SPA and LPA smooth muscle cells. ATP acts via a suramin-sensitive, PPADS-insensitive site, which most resembles the P2Y_11 _receptor. The site of action of UTP and UDP is less clear. Its pharmacology is inconsistent with our present understanding of P2Y_2,4 & 6 _receptors, so a novel receptor or receptor complex may be involved. These different P2Y receptors are likely to play a significant role in nucleotide-induced pulmonary vasoconstriction as ATP, UTP and UDP each induce contractions of the rat pulmonary artery with matching pharmacological profiles.

## Competing interests

The author(s) declare that they have no competing interests.

## Authors' contributions

KC was involved in the planning of the experiments described and carried them out. She also analysed the data, drafted the manuscript and was involved in its revision. AMG was involved in the planning of the experiments and revision of the manuscript. CK was involved in the planning of the experiments, the analysis of the data and revision of the manuscript. All authors read and approved the final manuscript.
